# Intra-species sialic acid polymorphism in humans: a common niche for influenza and coronavirus pandemics?

**DOI:** 10.1080/22221751.2021.1935329

**Published:** 2021-06-14

**Authors:** Xi Jiang, Ming Tan, Ming Xia, Pengwei Huang, Michael A. Kennedy

**Affiliations:** a Division of Infectious Diseases, Cincinnati Children's Hospital Medical Center, Cincinnati, OH, USA; b University of Cincinnati College of Medicine, Cincinnati, OH, USA; c Department of Chemistry and Biochemistry, Miami University, Oxford, OH, USA

**Keywords:** Influenza and coronavirus, 1918 H1N1 pandemic, sialic acid receptor, polymorphism, co-evolution

## Abstract

The ongoing COVID-19 pandemic has led to more than 159 million confirmed cases with over 3.3 million deaths worldwide, but it remains mystery why most infected individuals (∼98%) were asymptomatic or only experienced mild illness. The same mystery applies to the deadly 1918 H1N1 influenza pandemic, which has puzzled the field for a century. Here we discuss dual potential properties of the 1918 H1N1 pandemic viruses that led to the high fatality rate in the small portion of severe cases, while about 98% infected persons in the United States were self-limited with mild symptoms, or even asymptomatic. These variations now have been postulated to be impacted by polymorphisms of the sialic acid receptors in the general population. Since coronaviruses (CoVs) also recognize sialic acid receptors and cause severe acute respiratory syndrome epidemics and pandemics, similar principles of influenza virus evolution and pandemicity may also apply to CoVs. A potential common principle of pathogen/host co-evolution of influenza and CoVs under selection of host sialic acids in parallel with different epidemic and pandemic influenza and coronaviruses is discussed.

## Introduction

Influenza A viruses (IAVs) are widespread pathogens that cause annual seasonal epidemics and irregular, unpredictable pandemics with severe pathogenic effects and high fatality rates. Six IAV pandemics with three HA subtypes (H1, H2, and H3) have been documented in the past century, including the deadliest 1918 H1N1 pandemic that claimed ∼50 million lives globally. IAVs also infect wild and domestic animals with wild waterfowl as the natural reservoir. All known pandemic and other human, mammalian and poultry IAVs are believed to be descended from wild-bird viruses. It is also known that all native avian and many animal IAVs prefer binding sialic acids (Sias) linked to galactoses with an α2,3-linkage (Siaα2,3Gal), whereas human-adapted IAVs prefer the α2,6 linkage (Siaα2,6Gal). However, the application of these rules derived from research of influenza disease and epidemiology to date has been unable to explain many unusual epidemics, the high fatality pattern of the 1918 H1N1 pandemic [1–6], and other influenza pandemics in the past. This knowledge gap in understanding of major pandemics, such as the 1918 H1N1 pandemic, as well as the current COVID-19 pandemic, complicates development of strategies to control and prevent future influenza epidemics and pandemics.

Here we discuss a dual mutational mechanism of influenza virus evolution in humans that has resulted in two human H1 subtypes, a human-like H1 D225 subtype and an avian-like H1 225G subtype, each with distinct transmissibility and pathogenicity. We argue that the H1 D225 subtype caused the widespread transmission, while the H1 225G subtype caused high fatality in a small subpopulation of the 1918 H1N1 pandemic, and both subtypes also caused H1N1 seasonal epidemics but with much lower epidemic scales. Specifically, we postulate that the well-adapted human-like H1 D225 subtype originated from an avian virus [7] that adapted to humans by recognizing the 2,6-Sias in the upper respiratory tracts of the general population, which consequently led to high transmissibility and is therefore responsible for the widespread nature of both seasonal epidemics and pandemics with mild illness. On the other hand, the avian-like 225G subtype is descended from the human D225 subtype, which infected a small subset of the human population with a dominant 2,3-Sia in their lower respiratory tracts that resulted in limited transmissibility but led to severe cases with high fatality rates due to lower respiratory tract infections, resulting in pneumonia, which is more common in pandemics. Indeed, quasispecies with D225 and 225G subtypes of the hemagglutinin (HA) genes have been identified in archived specimens of the 1918 H1N1 pandemic victims [7–9] representing a valuable discovery.

Elucidation of the dual subtypes explains both the widespread but mild epidemic cases that occur in the majority of the human population that express 2,6-Sias and the severe cases that occurs in the minority of the same human population with polymorphic 2,3-Sias. This would also help explain pandemic threats posed by other emerging avian HA subtype viruses, such as the H5N1 and H7N9 subtypes. These emerging avian viruses cause usually sporadic epidemics with high fatality rates but with limited human transmission likely only involving in the small subpopulation with the 2,3-Sias in their lower respiratory tracts and thus may represent low pandemic risks. Since coronaviruses (CoVs) also recognize Sias as host receptors [10–12], this new knowledge regarding influenza viruses may help the understanding of CoV-caused epidemics and pandemics under selection of polymorphic human Sia receptors, such as the ongoing COVID-19 pandemic.

### The H1 D225 subtype may be responsible for the widespread nature and mild cases of the 1918 H1N1 pandemic

This hypothesis is based on a bidirectional concerted evolution of humans with an upregulation of the 2,6-Sia expression in the upper respiratory tracts of humans as shown by Sia signals in different cell types and body parts of humans compared with the great apes (chimpanzee, bonobo, gorilla, and orangutan) [13]. Considering the facts that the great apes are closest relatives of humans, sharing 97–99% of DNA sequence identity, and that chimpanzees are naturally resistant to the human 1918 H1N1 subtype recognizing the 2,6-Sias [13, 14], it is assumed that the dominant 2,6-Sias in the upper respiratory tracts of modern human populations could be a result of human co-evolution in an arms-race of our ancient hominid ancestors against the deadly avian IAVs or other 2,3-Sia binding pathogens over the past 100–200 thousand years [13], which possibly experienced multiple selective swipes driven by Sia-binding pathogens.

On the other hand, the emergence of the H1 D225 subtype in humans could also have been a countermeasure of the deadly avian viruses that switched from “avian-like” to a “human-like” Sia-binding properties under selection of the 2,6-Sias in humans in the upper respiratory tracts, resulting in widespread mild cases in both seasonal epidemics and pandemics. Phylogenetic analyses of the 1918 H1 pandemic viruses showed wide variations of their HA sequences compared with their closest avian relatives [9]. This indicates that complicated mutational mechanisms were involved in evolution and adaptation required for a native avian influenza strain to infect humans [7,15]. This process may have involved complex gene constellations, explaining why only three HA subtype (H1, H2 and H3) from the 18 known avian HA subtypes had successfully adapted to humans and became human endemic. This scenario could also explain why many avian HA subtypes that emerge in humans cause severe epidemics with high fatality rates mainly in the small subpopulation of humans who express 2,3-Sias in their lower respiratory tract. Avian H5N1 and H7N9 subtypes are such examples, usually with limited human to human transmissibility and therefore they represent low risks of pandemic threats (see below). Finally, since a few animal species, such as domestic pigs and ferrets, also express 2,6-Sias in their upper respiratory tracts, it would not be surprising to detect 1918 H1 viruses in these animal species, suggesting that the 2009 H1N1 pandemic could have originated in humans without a need of an animal intermediator.

### The H1 225G subtype viruses are responsible for the severe pathogenicity and high fatality of the 1918 pandemic

**Figure 1.**
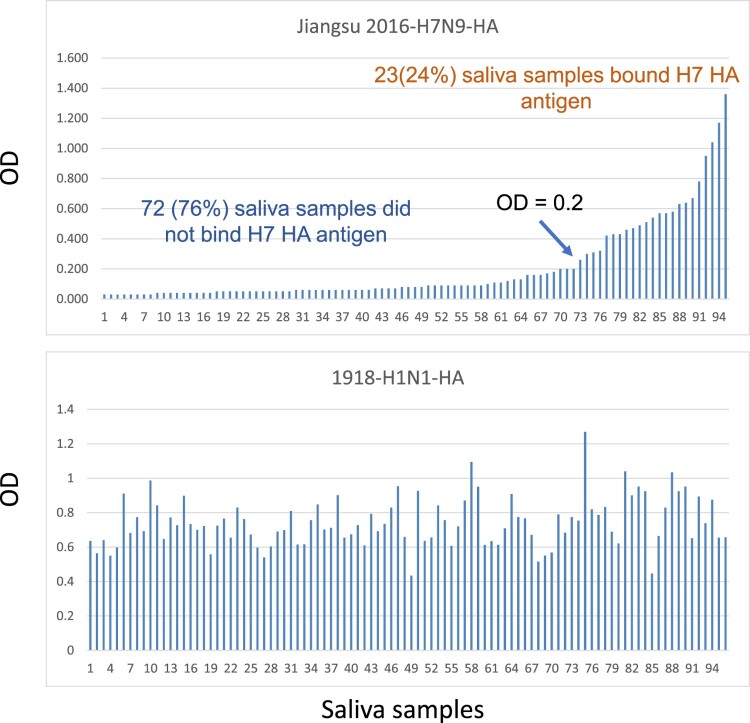
Binding assays of H7N9 and 1918 H1N1 HAs with saliva samples from a panel of health adults. Saliva samples from healthy employees in the Cincinnati Children’s Hospital Medical Center collected for studies of norovirus and rotavirus binding profiles of host histo blood group antigens in our previous studies [17,18] were used. Recombinant H7N9 and 1918 H1N1 HAs were constructed and expressed as described previously [16]. The 95 tested saliva sample donors were sorted based on the OD binding signals of H7N9 HA (top panel).

**Figure 2.**
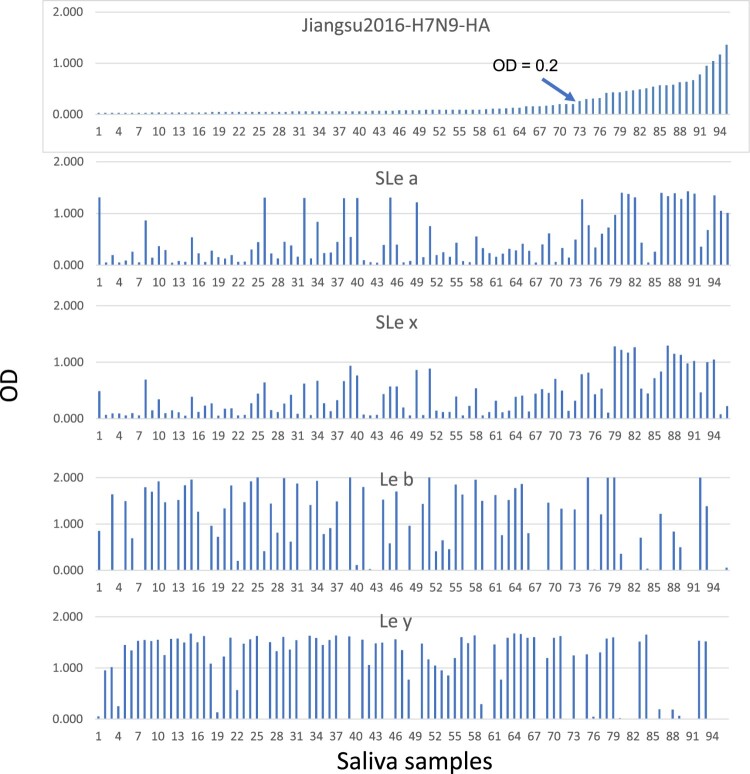
Saliva binding profiles of H7N9 HA in association with the sialic acid types of saliva donors. The binding signals of sialic Lewis a (SLe a), sialic Lewis x (SLe x), Lewis b (Le b) and Lewis y (Le y) in the saliva samples were performed ELISA using commercial monoclonal antibodies described previously [13]. The 95 tested saliva sample donors were sorted based on the OD binding signals of H7N9 HA (top panel).

**Figure 3.**
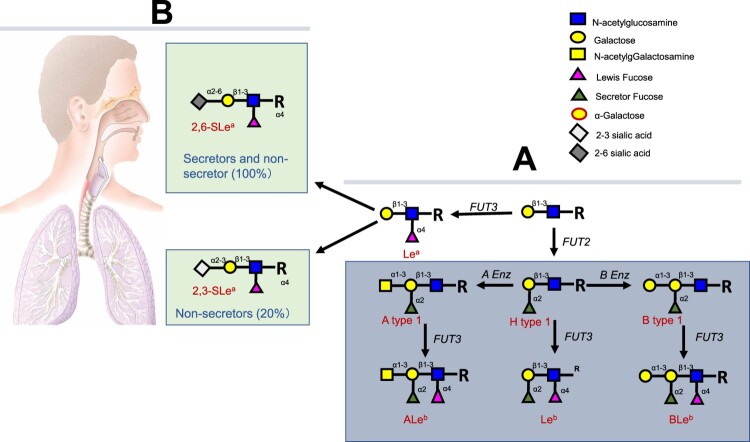
Synthesis of the ABH and Lewis histo-blood group antigens and deduced blocking or masking of synthesis of 2,3-sialic acid antigens by preoccupied 1,2-linked fucose by the H fucose-transferase encoded by the FUT 2 gene in humans. (A) Biosynthesis of type 1 based HBGAs. Synthesis proceeds by stepwise addition of monosaccharide units from a precursor disaccharide present at the terminus of glycan chains from either O-linked or N-linked glycans of glycoproteins, or from glycolipids (R) [19]. (B) Deduced blocking or masking of synthesis of the 2,3-sialic acid antigens by preoccupied 1,2-linked fucose synthesized by the H fucose-transferase encoded by the FUT 2 gene in humans following a study of H7N9-infected patients involved in an outbreak in China in season 2016/17. Around 80% of the general populations are secretor positive and the expression of the 2,3-linked sialic acids are blocked or masked by the preoccupied 1,2-linked fucose, suggesting that secretors may be naturally resistant to H7N9 IAVs because they mainly express the 2, 6-linked sialic acids in the upper respiratory tracts and may not express or express low amounts of 2,3-linked sialic acids in their lower respiratory tracts. The 20% non-secretors may also express 2,6-linked sialic acids in the upper respiratory tracts but mainly express the 2,3-linked sialic acids in the lower respiratory tracts.

We first formulated the hypothesis of polymorphic 2,3-Sias in humans after finding that only ∼30% of human subjects revealed ELISA binding signals in their saliva specimens by a recombinant HA of the avian H7N9 viruses that bind 2,3- but not 2,6-linked Sias [16]. Our further studies of saliva specimens from a panel of healthy adults confirmed the results with ∼24% of the saliva panel binding to the H7 HA antigens but, by comparison, 100% of the saliva panel revealed binding signals with a recombinant 1918 H1 antigen that is known to recognize 2,6-Sias (Figure 1). The saliva H7 binding profiles correlated with the secretor status of the saliva sample donors and reciprocal correlations were observed between secretors and non-secretor donors (Figure 2). Around 80% of the general population are secretors and ∼20% are non-secretors in the North America and European countries. The saliva donors’ secretor types are controlled by the *FUT2* gene encoding a fucose-transferase responsible for synthesis of fucose containing oligosaccharides in the ABO, Lewis and secretor (H) histo-blood group antigen (HBGA) families, in which the FUT2 fucose transferase catalysing the addition of a fucose with a 1,2-linkage to the galactose (Figure 3). Thus, lack of saliva binding by the H7 HA antigen in the majority secretor donors is a result of the preference of the H7 HA antigen to 2,3-Sias. The synthesis of the 2,3-Sias is blocked in the majority secretor population due to steric interference by the 1,2-linked fucose, and this leads to the sterically allowed synthesis of the 2,6-Sias in the majority secretor population. This observation leads to a hypothesis of dual causes of influenza pandemics with a human-like H1 subtype infecting most of the human population with dominant 2,6-Sias in their upper respiratory tracts responsible for the high transmissibility and widespread nature and another avian-like H1 subtype infecting a small subset of human population with a dominant 2,3-Sias in the lower respiratory tracts that were responsible for the severe cases and high fatality rates of the 1918 pandemic.

The scenario of dual causes of influenza epidemics and pandemics also explains the unusual epidemic patterns observed during the 1918 H1N1 pandemic. For example, three epidemic waves occurred in the 1918 H1N1 pandemic with a much more severe fall of 1918 wave compared to the 1918 spring wave that could have been due to a low start of the circulating 225G subtype in the spring wave because the 225G viruses may have suffered low survival during the off-season and needed time to build up circulation by continual accumulation with the new emerging 225G variants over the course of the pandemic. On the other hand, the high mortality rates of young adults in the 1918 pandemic could have been due to a lack of pre-existing antibodies against the D225 H1 and 225G subtypes due to dominant H3N2 pandemics and epidemics [20] during their childhood resulting in the W-shaped age mortality compared with other age groups [1,2,20,21], although increased mortality rates of young adult soldiers in World War I could also have contributed to the W shape of age-specific fatalities. Furthermore, the reason why the elderly populations had a relative lower mortality rate than expected could also be due to their exposures in early life and therefore they may have obtained immunity against the H1N1 subtype viruses. This was demonstrated by the detection of protective antibodies against the 1918 H1N1 subtype viruses in seniors who survived the 1918 pandemic [22, 23].

Mutant H1 D225G subtype viruses also have been identified in clinical specimens from the 2009 H1N1 pandemic [24, 25]. Such H1 225G variants were detected more frequently in specimens collected from the lower respiratory tracts than in the upper respiratory tracts in severe cases and in cases with serious clinical outcomes and death [24]. Mutant 225G HAs bind a broader range of 2,3-linked secretor sequences of a type expressed on ciliated bronchial epithelial cells and on epithelia within the lung [26]. Evidence of D225G mutant viruses arisen *de novo in vivo* has also been suggested by a study of an immunocompromised human case by comparison of 225G variants that appeared in specimens harvested at different stages of clinical illness and in ferret models [27] following challenge of mixed D225/225G inoculum or cloned 225G isolates [27]. These findings support the idea that the 2009 H1N1 pandemic subtype is a descendent of the D225 subtype of the 1918 pandemic with a newly emerging H1 225G subtype arisen *de novo* in humans and triggered or strengthened the 2009 pandemic with or without an animal intermediator. Thus, while the D225 subtype is well-adapted to humans and causes upper respiratory tract infections with mild illness in the general human population expressing the 2,6-Sias, the 225G subtype mainly causes severe lower respiratory tract infections and pneumonia in the subpopulation with dominant 2,3-Sias in their lower respiratory tracts and is mainly responsible for the observed high fatality rates. This principle may apply to all H1N1 caused pandemics that have occurred in the past century and similar cycles of seasonal epidemics and occasional pandemics that are expected to continue.

### Impact on disease control and prevention of influenza epidemics and pandemics

The new understanding of influenza virus evolution under selection of polymorphic Sia receptors in humans presented here helps explain many heretofore unanswered questions concerning the unusual epidemic patterns and high fatality rates of influenza pandemics. This new knowledge may impact future vaccine development against influenza epidemics and pandemics. For example, since the well-adapted 1918 human H1N1 subtype is the mainstream of viruses causing both seasonal infections and pandemics and is under strong positive selection by the dominant 2,6-Sias in the general human population, a vaccine targeting the antigenic determinants in the HA receptor binding domains may provide long lasting protection to avoid annual booster vaccinations. On the other hand, immune responses to conserved antigenic determinants on HA heads outside the receptor binding domains may also occur and lead to lifelong positive or negative immune effects, known as “antigen imprinting” or “antigenic seniority” [28–30], which also need to be considered in new vaccine development. Future studies to develop highly sensitive and specific assays to measure Sia receptor-specific blocking or neutralizing antibody responses for vaccine efficacy assessment are important and necessary.

The elucidation of the origins and evolutionary paths of different seasonal/pandemic viruses and the new emerging zoonotic avian HA subtypes should also help guide research and strategy development to predict and prepare against future influenza pandemics. For example, the human H1N1 subtype is well adapted possibly by one entrance and became human endemic, thus, the annual seasonal epidemics and occasional pandemic cycles could continue into the future, which may or may not need an animal intermediator. On the other hand, cross-species transmission of many newly emerging avian influenza HA subtypes into humans was permissible to only a small subpopulation expressing the 2,3-Sias, such as the emerging H5N1 and H7N9 subtypes, and these viruses may not pose pandemic threats. Thus, while an avian vaccine is important for protection of the poultry industry, a human vaccine may not be necessary; instead a screening for 2,3-Sia positive individuals and prohibit them to work with poultry may be an effective strategy. Establishment of Sia-type identity in the general population may also help disease control and prevention against many other Sia-recognized viral pathogens.

### The same principle of influenza virus evolution under selection of polymorphic 2,3-Sias in humans may also apply to coronavirus-caused epidemics and pandemics

Based on the new understanding of influenza virus evolution under control of the polymorphism of 2,3-linked Sias in humans, we propose that the same principle may also apply to CoV-caused epidemics and pandemics because CoVs also recognize Sia receptors [11, 12, 31–33]. CoVs also infect wild and domestic animals with wild bats as the natural reservoir and known human and many mammalian CoVs are believed to be descended from bats. It is also known that some animal CoVs prefer binding Sias linked to galactose with a α2,3-linkage (Siaα2,3Gal), however, at least one human-adapted CoV subtype, the human-adapted HCoV-OC43, prefers the α2,6 linkage (Siaα2,6Gal) [12]. By comparison of the seven CoV strains that emerged in humans in the past century (Table 1), we propose a convergent evolutionary relationship between CoVs and influenza viruses under selection of Sia receptors as a common niche, with individual sub-lineages emerging in parallel between the two viral species and causing subtype-specific seasonal epidemics and pandemics.

**Table 1.** Deduced sialic acids and their glycan linkages as receptors for selected human and zoonotic IAVs and CoVs.

Virus	Receptor	Glycan linkage	Clinical infections and epidemics
**IAVs**			
H1N1	Neu5Ac-Sias	α2,6-linkage*	Seasonal and pandemic flu, 1918, 2009
H2N2	Neu5Ac-Sias	α2,6-linkage	Seasonal and pandemic flu 1957
H3N2	Neu5Ac-Sias	α2,6-linkage	Seasonal and Pandemic flu 1968
H5N1	Neu5Ac-Sias	α2,3-linkage?	Avian flu 1997–
H7N9	Neu5Ac-Sias	α2,3-linkage	Avian flu 2013–
**CoVs**			
HCoV-OC43	9-O-AC-Sias	α2,6-linkage	Endemic in human, mild respiratory tract infections
HCoV-HKU1	9-O-AC-Sias	α2,6-linkage?	Endemic in human, mild respiratory tract infections
HCoV-NL63	9-O-AC-Sias	α2,6-linkage?	Endemic in human, mild respiratory tract infections
HCoV-229E	9-O-AC-Sias	α2,6-linkage?	Endemic in human, mild respiratory tract infections
SARS-CoV	Neu5Ac-Sias	α2,3-linkage?	Severe acute respiratory syndromes 2002–2003
SARS-CoV2	Neu5Ac-Sias	α2,6-linkage?*	Severe acute respiratory syndromes 2019–
MERS-CoV	Neu5Ac-Sias	α2,3-linkage	Middle East respiratory syndromes 2012–2014

Notes: ? Indicating deduced sialic acids and types of glycan linkages based on our preliminary studies and rationale literatures. * Indicating also recognizes α2,3-linkage.

For example, the four strains of human CoVs, HCoV-NL63, HCoV-229E (α-coronaviruses) and HCoV-OC43 and HCoV-HKU1 (β-coronaviruses) that are endemic, i.e. well-tolerated in humans, and account for up to 30% of mild respiratory tract infections, recognize 9-O-AC modified sialic acids (9-O-AC-Sias) with 2,6-linkages (Table 1) [12]. This situation is similar to the seasonal influenza C viruses (ICVs) that also recognize 9-O-AC-Sias with 2,6-linkages and only cause mild infections associated with the common cold. In addition, the Middle East respiratory syndrome coronaviruses (MERS-CoVs) that emerged in the Arabian Peninsula in 2012, which caused recurrent sporadic outbreaks in humans with a fatality rate of 35%, were introduced to humans via domestic dromedary camels and caused only sporadic cases until 2017 with limited human-to-human transmission [11, 12]. This situation is very similar to the zoonotic avian H7N9 IAVs that were transmitted to humans through domestic poultry and caused sporadic cases in outbreaks for five seasons in China since 2012 before being contained in 2017 [34]. Interestingly, both MERS and H7N9 recognize unmodified sialic acids (Neu5Ac-Sias) with 2,3-glycan linkages [11] (Table 1) and are of animal origins. This may explain their limited human-to-human transmission and sporadic cases with severe illness and high human fatality rates, which may again be due to recognition of the less common 2,3-linked Sias in lower respiratory tracts that occur in just ∼20% of the human population. Similarly, the SARS-CoV that emerged in 2002 and was responsible for an epidemic that spread to five continents with a fatality rate of ∼10% before being contained in 2003, could also be explained by the same mechanism of cross-species transmission as occurred in the MERS CoVs.

On the other hand, the continuation of the COVID-19 pandemic caused by SARS-CoV-2 is very similar to the 1918 H1N1 pandemic, and therefore may share a similar principle of a zoonotic source and a cross-species pathway for transmission as occurred in the 1918 H1N1 influenza pandemic that was widespread due to high transmissibility and had a high fatality rate. Limited evidence supporting this hypothesis includes the finding that the SARS-CoV-2 recognizes non-modified Sias [35, 36] consistent with their close genetic relatedness with the SARS- and MERS-CoVs [36–38] that also recognize non-modified Sias (for MERS-CoVs, [39]). Thus, we propose that these three CoVs may have a common ancestor and share similar evolutionary pathways of serial mutations and adaptations that occurred with the influenza seasonal and pandemic viruses, but with two new CoV subtypes; one with a gained human-like 2,6-Sia binding property with high transmissibility responsible for the asymptomatic and mild cases occurring in the majority secretor general population, and the other subtype with an animal 2,3-Sia binding property and mainly circulating in the small non-secretor subpopulation with dominant 2,3-Sias in their lower respiratory tracts, but that has been responsible for the severe cases and high fatality rate seen in the COVID-19 pandemic. Like the 1918 H1N1 pandemic, the deduced new CoV subtypes also may have existed in humans for some time, and possibly was responsible for several low-level seasonal epidemics before the current pandemic surfaced in the fall of 2019. An estimated 2–15 year pre-pandemic emergence period has been suggested for the occurrence of the pandemic in 1918 [7, 15], suggesting that COVID-19 pandemic viruses could also have existed in humans after the initial cross-species introduction to humans and underwent multiple low-level seasonal epidemics before the occurrence of the pandemic in the fall of 2019. Thus, due to the expected timeline required for pre-pandemic emergence, it is highly unlikely that the SARS-CoV-2 viruses were originated from laboratory manipulation, rather than by natural selection in humans following zoonotic transfer.

Finally, CoVs also recognize protein receptors, with the SARS-CoV-2 recognizing the host angiotensin-converting enzyme 2 (ACE2) via the viral spike proteins and employ the cellular serine protease TMPRSS2 for priming as the major cellular entry point. Variations in expression of these host proteins could influence the entry of SARS-CoV-2 viruses into the host cells and thus affect the transmissibility and even severity of the disease. In addition, variability in innate immune system components among humans also may contribute to the heterogenous disease courses of COVID-19 patients [40] which also need to be considered in clinical and epidemiological assessments for strategy development in control of the COVID-19 and prevention of future CoV epidemics and pandemics.

## Conclusion and perspective

The elucidation of the polymorphic Sias in the modern human population significantly improved our understanding on the widespread natures and high fatality in only a small subpopulation of influenza epidemics and pandemics which will impact future strategy for influenza disease and epidemic control and prevention. Vaccines targeting the highly conserved antigenic determinants in the receptor binding domains on the HA head to the unanimous 2,6-Sias in the general population would likely induce long-lasting immune protection without the need of the annual booster. The deduced long-term immune protection may help the reassessment of the “original antigen sin” or “antigen imprinting” phenomenon for better understanding of adaptive immunity against influenza viruses for better vaccine designs. New understanding of the emerging avian influenza H5N1 and H7N9 subtype-caused epidemics in only a small subpopulation with dominant 2,3-Sias may provide the means to develop a rational strategy for epidemic control and prevention in the poultry industry and targeted human protection of the susceptible individuals. The same principles of evolution, disease patterns, and epidemiology of influenza viruses may also apply to CoVs with possible similar strategies between the SARS-CoV-2 and influenza H1N1 and between SARS-/MERS-CoVs and H5N1/H7N9, respectively.

Our hypothesis to explain influenza and coronavirus evolution and pandemicity remains preliminary particularly for the CoV-caused epidemics and pandemics and further studies are required to collect solid evidence to support our hypothesis. The most important study is to verify the association of susceptibility and severity of illness in CoV infected patients with their genetic and phenotypic makeup of the 2,6- vs. 2,3-Sias in their upper vs. lower respiratory tracts through large population studies of CoV-infected patients with different clinical outcomes in different countries and ethnic populations. In addition, determination of the distributions of the 2,3- and 2,6-Sias in different ethnic populations and age groups in different countries of the world population will be important to guide strategy and policy-making decisions made to control and prevent influenza and coronavirus-caused epidemics and pandemics in the future. These studies are also important for understanding many other viral and microorganism pathogens that also recognize Sia receptors [41–44].
